# Interleukin-31 in serum and cerebrospinal fluid of dogs with syringomyelia

**DOI:** 10.1186/s12917-023-03817-8

**Published:** 2023-11-23

**Authors:** Laura Lemke, Regina Carlson, Thomas Flegel, Andrea Volk, Holger Andreas Volk, Andrea Tipold, Jasmin Nicole Nessler

**Affiliations:** 1 Department of Small Animal Medicine and Surgery, University of Veterinary Medicine Hannover, Hannover, Germany; 2 Department of Small Animal Medicine, Leipzig University, Leipzig, Germany

**Keywords:** Interleukin-31, Syringomyelia, IL-31, Air-scratching, Cerebrospinal fluid, Serum

## Abstract

**Background:**

Syringomyelia is a spinal cord cavity containing cerebrospinal fluid (CSF)-like fluid. If syringomyelia asymmetrically involves the dorsal horn grey matter of the spinal cord, affected dogs show increased signs of dysesthesia and neuropathic pain, like increased itching behaviour. In the dorsal horn, amongst others, receptors for Interleukin-31 (IL-31) can be found. IL-31 is one of the main cytokines involved in the pathogenesis of pruritus in atopic dermatitis in different species. This study investigates suspected elevated levels of IL-31 in serum and CSF of dogs showing signs of pain or increased itching behaviour related to syringomyelia. The IL-31 were measured in archived samples (52 serum and 35 CSF samples) of dogs with syringomyelia (n = 48), atopic dermatitis (n = 3) and of healthy control dogs (n = 11) using a competitive canine IL-31 ELISA.

**Results:**

Mean serum IL-31 level in dogs with syringomyelia was 150.1 pg/ml (n = 39), in dogs with atopic dermatitis 228.3 pg/ml (n = 3) and in healthy dogs 80.7 pg/ml (n = 10). Mean CSF IL-31 value was 146.3 pg/ml (n = 27) in dogs with syringomyelia and 186.2 pg/ml (n = 8) in healthy dogs. Individual patients with syringomyelia (especially dogs with otitis media or otitis media and interna or intervertebral disc herniation) showed high IL-31 levels in serum and CSF samples, but the difference was not statistically significant. IL-31 serum and CSF levels did not differ significantly in dogs with syringomyelia with or without itching behaviour and with or without signs of pain.

**Conclusion:**

Based on this study, increased IL-31 levels seem not to be correlated with itching behaviour or signs of pain in dogs with syringomyelia, but might be caused by other underlying diseases.

**Supplementary Information:**

The online version contains supplementary material available at 10.1186/s12917-023-03817-8.

## Background

Itching or pruritus is an unpleasant feeling which is mostly answered by the affected individuum by scratching the affected skin area [1]. Several reasons for pruritus are known and can be grouped amongst others into primarily skin derived, neuropathic, which is caused by pathological changes of sensory nerve fibres, and neurogenic, which is caused by mediators and receptors in the central nervous system without direct neuronal damage [1]. In atopic dermatitis pruritus is mediated among others via elevated Interleukin-31 (IL-31) which is produced by T-helper (Th) 2 cells [2–9]. IL-31 develops its effect via binding to a receptor complex of the IL-31 receptor A (IL-31RA) and the oncostatin M receptor β (OSMR) [3, 4, 7, 9]. IL-31RA can be detected on sensory neurons in the dorsal root ganglion and in the outer lamina II of the dorsal horn of the spinal cord [6, 10], where it mediates the transmission of the pruritic signal via pruritogen-sensitive neurons [11]. This pathway overlaps with the nociception pathway within the dorsal horn [8, 11, 12].

**Fig. 1:**
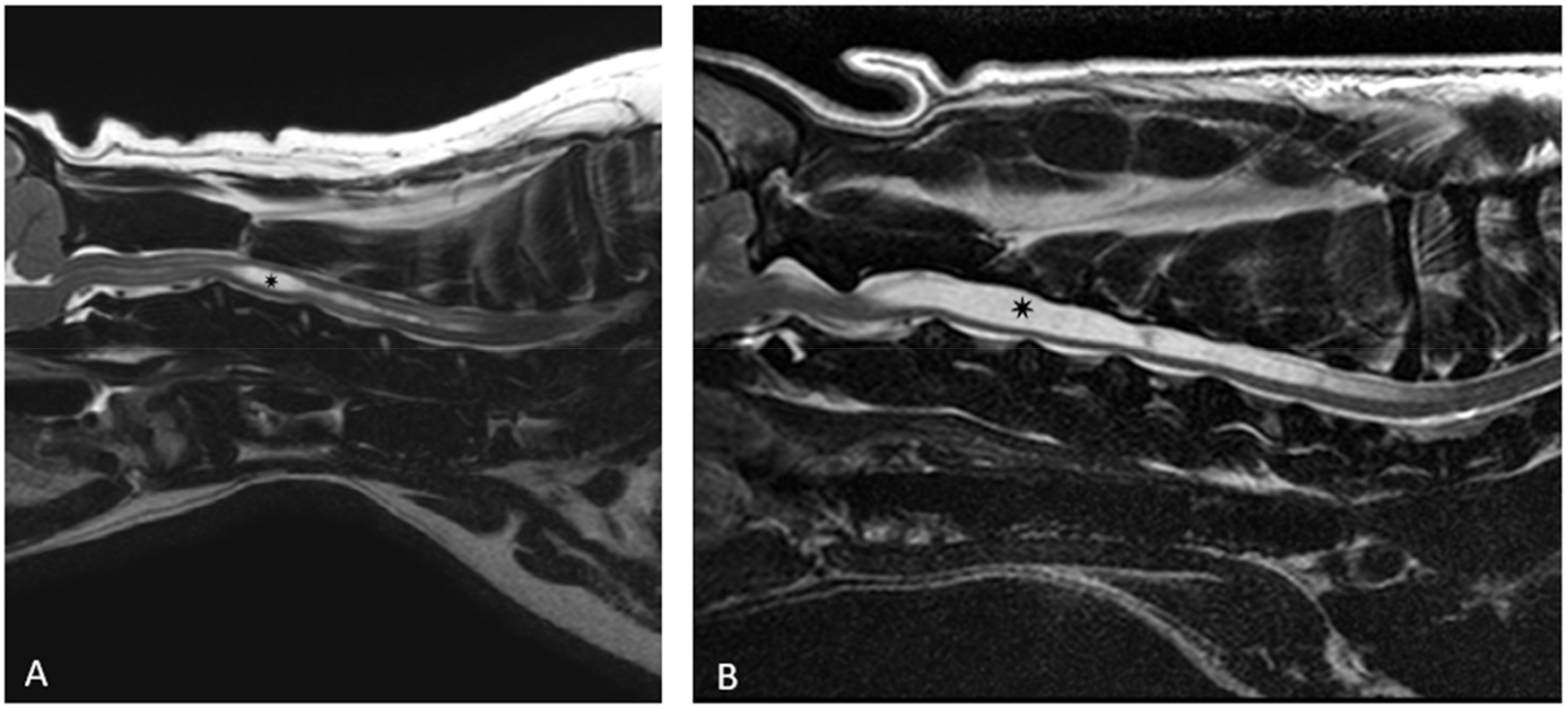
(**A**) and (**B**): T2-weighted sagittal magnetic resonance images of the cervical spinal cord of two different dogs with syringomyelia which appears T2-hyperintense in comparison to the spinal cord and cerebrospinal fluid-isointense (marked with a star). Figure 1A represents a focal, moderate syringomyelia, pronounced at the level of C3. Figure 1B displays a more pronounced case of a syringomyelia extending from the level C2 to T1 and involving the dorsal horn

A high frequency of itching behaviour and signs of pain can be observed in dogs with syringomyelia [13]. Syringomyelia describes extensive spinal cord cavities which contain cerebrospinal fluid (CSF)-like fluid (Fig. 1) [14]. Syringomyelia is suspected to be the consequence of abnormal CSF flow [14] and frequently occurs in connection with calvarian malformation [13–15] or secondary to other structural changes in the brain or spinal cord, including, but not limited to neoplasia [16, 17], trauma, inflammation, or degenerative processes [18]. Clinical signs like signs of dysesthesia and pain can be aggravated or provoked when touching the head or neck [13, 14]. Another clinical sign of syringomyelia is so-called air scratching or phantom scratching [13, 14], which is visible in 28% of the affected dogs [15]. This is characterized by non-purposeful rhythmic scratching movements, with no visible skin contact of the ipsilateral pelvic limb [15, 19]. The exact pathomechanism has not been fully clarified yet [19]. The previous literature is somewhat controversial if the occurrence of signs of pain or itching behaviour is correlated with the size of the cavity [14, 20, 21]. However, dorsal location of the syrinx was correlated with pain and scratching behaviour [21] and additionally Nalborczyk et al. assumed that syringomyelia affecting the dorsal horn of the spinal cord segments C3-6 leads to an increased occurrence of air scratching [19].

Since IL-31RA occurs in the dorsal horn of the spinal cord, we hypothesize that dogs with syringomyelia and signs of pain or itching behaviour have an elevated level of IL-31 in CSF and/or serum.

In this retrospective, multicentre study, we included dogs with magnetic resonance imaging (MRI) confirmed syringomyelia with or without neurological comorbidities and examined serum and CSF IL-31 levels. Dogs with neurological comorbidities suffered from intervertebral disc extrusions or neoplasia confined to the cervical vertebral column or spinal cord. Healthy dogs and dogs with atopic dermatitis served as control groups. Increased itching behaviour, signs of pain, and other comorbidities which might cause itching behaviour or signs of pain were documented. For detailed information see paragraph “material and methods”.

## Results

Single CSF or serum samples had to be excluded as coefficient of variation was > 20%. Therefore, for single dogs, only IL-31 levels of CSF or serum are available for statistical analysis. A total of 52 serum and 35 CSF samples from 62 patients could be used for the statistical evaluation. The sample distribution was as follows:

- 48 patients with syringomyelia (group A).
- 3 patients with atopic dermatitis (group B).
- 11 healthy control dogs (group C).

The group of patients with syringomyelia (group A) was further divided in three subgroups to exclude an influence of comorbidities on IL-31 levels in these patients: Patients with syringomyelia and no structural brain lesion (subgroup A.1, n = 27), patients with syringomyelia and otitis media and/or interna (subgroup A.2, n = 8; including primary secretory otitis [n = 4] and infectious otitis media and interna [n = 4]) and patients with syringomyelia and concomitant neurological disease (subgroup A.3, n = 13). In this group the syringomyelia was either an incidental finding or occurring secondarily to the underlying disease. Of these dogs seven dogs had an additional intervertebral disc herniation, three dogs had a suspected neoplasia, one dog each had either a suspected ischemic infarct or reactive seizures or a traumatic soft tissue lesion (Supplement 1). Cavalier King Charles Spaniel was the most frequently represented breed (n = 19/62), followed by Beagle (n = 13/62; including eleven healthy Beagles of group C), French Bulldogs, Chihuahuas (each n = 8/62) and Jack Russel Terriers (n = 5/62). Additionally, one sample from the following breeds were included: Pug dog, mixed breed dog, Boxer, Maltese dog, Miniature Schnauzer, German Shepherd, Chow Chow, Golden Retriever and Berger de Pyrénées. 37% of the dogs were male (n = 23/62) and 22.6% of the dogs were male-neutered (n = 14/62), in addition 21% of the dogs were female (n = 13/62) and 19.4% female-spayed (n = 12/62). Median age of all dogs was 67.4 months (n = 62, range 15 to 141 months).

### Serum samples

**Fig. 2:**
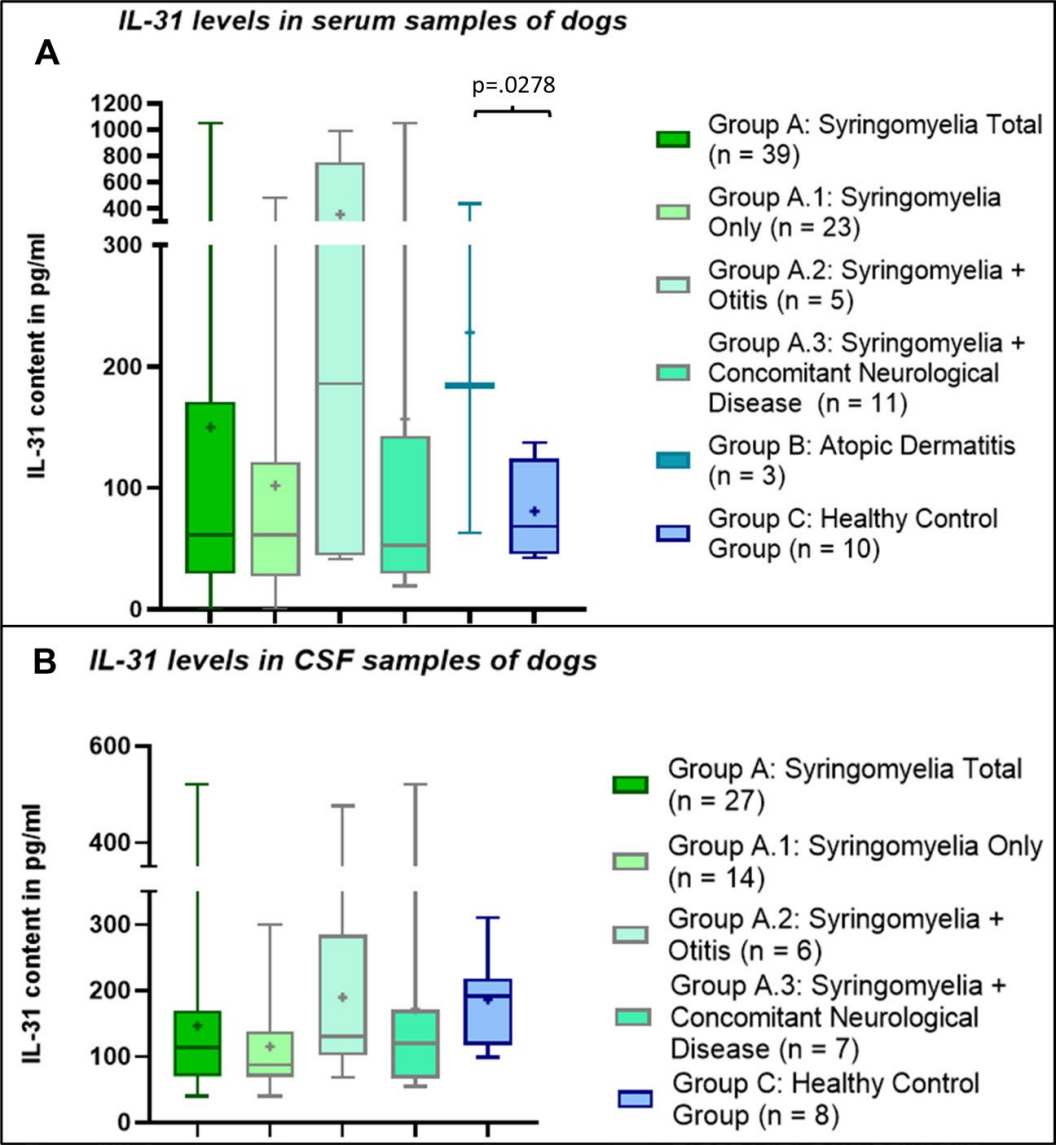
Interleukin-31 (IL-31) levels in serum and cerebrospinal fluid (CSF) samples of dogs. There was a significant difference within the serum samples between patients with atopic dermatitis (group B) and the samples from the healthy control group (group C) (p=.0278). The values of other groups did not differ significantly (p>.05). The boxplots show whiskers from the minimum to maximum and boxes from the 25th to 75th percentiles as well as the median (horizontal line) and the mean (+). pg/ml: picograms per milliliter

The mean IL-31 level in serum tended to be higher in patients with atopic dermatitis (group B), followed by patients with syringomyelia (group A) and the control group (group C) with the lowest mean IL-31 level (Table 1), but there was only a significant difference between the IL-31 level in the serum samples of patients with atopic dermatitis (group B) and the healthy control group (group C) (p = .0278, Fig. 2).

In subgroup A.2 with syringomyelia and otitis, the mean IL-31 level in serum tended to be higher compared to subgroups A.1 with syringomyelia only and A.3 with syringomyelia and other neurological diseases (Table 1), although this difference was not significant (p = .2263, Fig. 2).

In healthy control dogs the highest IL-31 level in serum was 137.2 pg/ml. In subgroup A.2 with syringomyelia and otitis (group A.2) three dogs (n = 3/5) had higher IL-31 levels in serum than the highest IL-31 level in healthy dogs (Table 1). Of the dogs with syringomyelia and concomitant neurological disease (group C) only dogs with intervertebral disc herniation (n = 3/11) had IL-31 levels in serum above the healthy control group (Table 1). An elevated IL-31 level > 1050 pg/ml was measured in the serum of one patient from group A.3 with syringomyelia and intervertebral disc extrusion. The exact value of this sample could not be determined as small sample size prevented repeated measurement of the diluted sample. Of the three dogs with atopic dermatitis (group B), two dogs had higher IL-31 levels in serum than healthy control dogs.

### CSF samples

The mean IL-31 levels in CSF did not differ significantly between groups A und C (p = .1028, Table 1; Fig. 2). No CSF samples from dogs with atopic dermatitis (group B) were included in this study.

Within the group of patients with syringomyelia (group A), IL-31 values did not differ significantly between the three subgroups A.1, A.2 and A.3 patients (p = .3151, Fig. 2) with highest IL-31 values in CSF measurable in patients with syringomyelia and additional otitis (group A.2).

In 25 dogs IL-31 levels in paired serum and CSF samples were available: 18 dogs with syringomyelia (group A) and seven dogs from the healthy control group (group C). In these dogs, IL-31 levels in serum samples were correlated with IL-31 levels in CSF (r = .5619; p = .0035).

**Table 1:** Descriptive statistics of Interleukin-31 (IL-31) levels in serum and cerebrospinal fluid (CSF) samples

Group		Type	Number	Mean IL-31 level (pg/ml)	Minimum IL-31 level (pg/ml)	Maximum IL-31 level (pg/ml)	Standard deviation
A	Syringomyelia total	Serum	39	150.1	0	> 1050	237.7
		CSF	27	146.3	40.35	521.5	119.0
A.1	Syringomyelia only	Serum	23	102.1	0	478.8	121.3
		CSF	14	115.1	40.35	300	75.09
A.2	Syringomyelia + otitis	Serum	5	356.5	41.29	986.2	402.5
		CSF	6	189.7	68.0	475.5	160.8
A.3	Syringomyelia + concomitant neurological disease	Serum	11	156.7	19.2	> 1050	301.3
		CSF	7	171.5	54.79	521.5	160.8
B	Atopic dermatitis	Serum	3	228.3	62.97	437.9	191.4
		CSF	-	-	-	-	-
C	Healthy control group	Serum	10	80.73	42.67	137.2	37.87
		CSF	8	186.2	98.63	309.6	186.2
**Total**		Serum	52	141.3	0	> 1050	212.1
		CSF	35	155.4	40.35	521.5	109.9

pg/ml: picograms per milliliter

### Itching behaviour

Of 48 dogs with syringomyelia 21 dogs showed itching behaviour (n = 10/27 with syringomyelia only, n = 7/8 with syringomyelia and otitis, n = 4/13 with syringomyelia and concomitant neurological disease), in two dogs no information regarding itching behaviour was available. All three dogs with atopic dermatitis and none of the 11 healthy beagles showed increased itching behaviour.

**Fig. 3:**
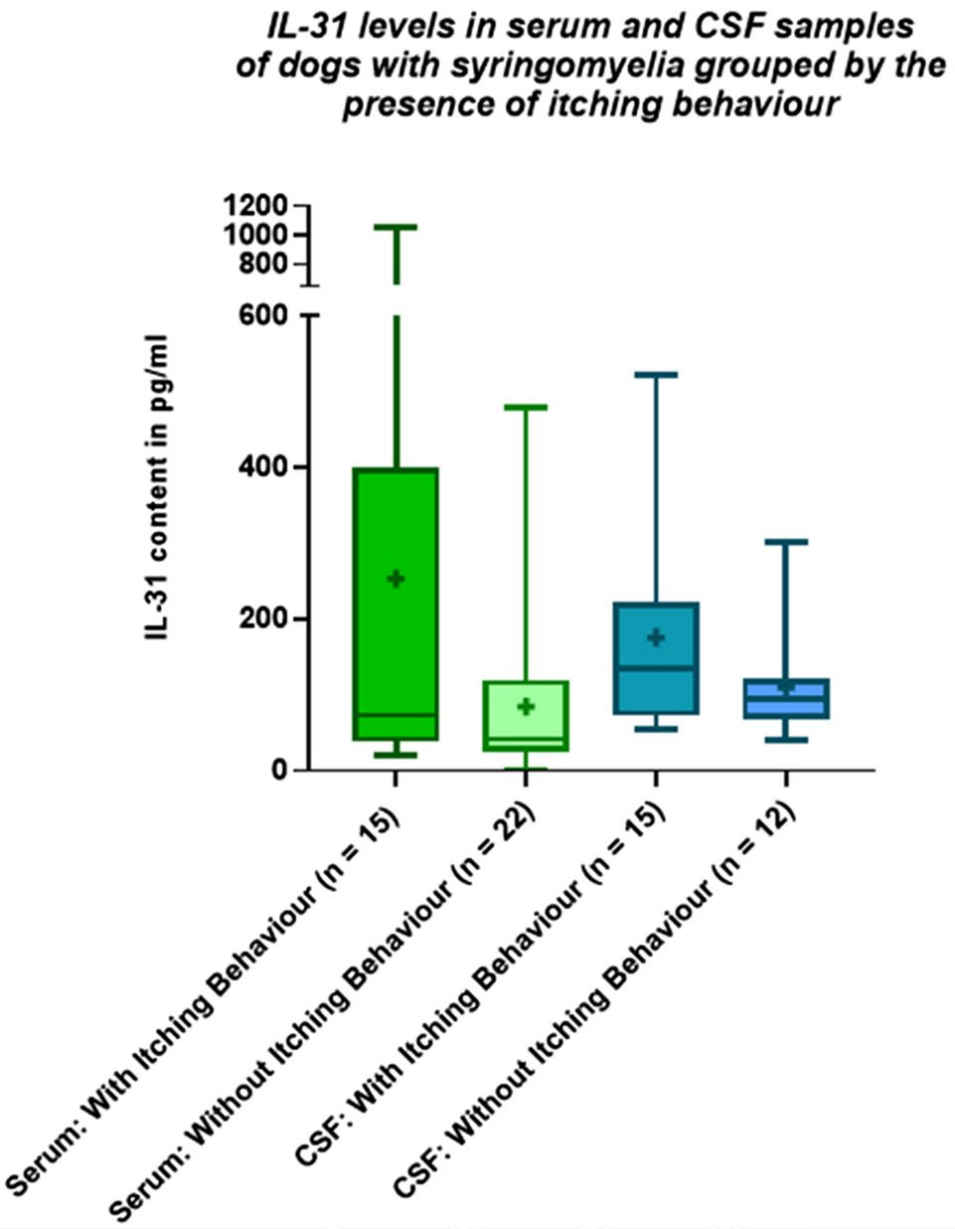
The Interleukin-31 (IL-31) levels in serum and cerebrospinal fluid (CSF) samples of dogs with syringomyelia (group A) grouped by their itching behaviour are shown in this figure. The boxplots show whiskers from the minimum to maximum and boxes from the 25th to 75th percentiles as well as the median (horizontal line) and the mean (+). No significant differences were detected between the groups, p > .05. pg/ml: picograms per milliliter

Dogs with syringomyelia displaying itching behaviour tended to have higher mean IL-31 level in serum and CSF compared to dogs with syringomyelia without itching behaviour or healthy control dogs (Table 2), although the results did not differ significantly in serum (p = .0607) and CSF (p = .1422, Fig. 3).

**Table 2:** Descriptive statistics of Interleukin-31 (IL-31) level in serum and cerebrospinal fluid (CSF) samples of dogs with syringomyelia considering the occurrence of itching behaviour

Group			Itching behaviour	Number	Mean IL-31 level (pg/ml)	Minimum IL-31 level (pg/ml)	Maximum IL-31 level (pg/ml)	Standard deviation
**Serum**								
A	Syringomyelia total		Yes	15	252.8	20.34	≥ 1050	342.9
			No	22	84.50	0	478.8	107.2
	A.1	Syringomyelia only	Yes	6	137.8	29.9	398.9	139.8
			No	15	87.82	0	478.8	123.8
	A.2	Syringomyelia + otitis	Yes	5	356.5	41.29	986.2	402.5
			No	0	/	/	/	/
	A.3	Syringomyelia + concomitant neurological disease	Yes	4	295.6	30.34	≥ 1050	503.4
			No	7	77.38	19.2	193.7	66.27
B	Atopic dermatitis		Yes	3	228.3	62.97	437.9	191.4
			No	/	/	/	/	/
C	Healthy control group		Yes	/	/	/	/	/
			No	10	80.73	42.67	137.2	37.87
**CSF**								
A	Syringomyelia total		Yes	15	175.4	54.79	521.5	143.1
			No	12	109.9	40.35	300.0	69.46
	A.1	Syringomyelia only	Yes	7	122.7	67.36	251.4	64.65
			No	7	107.4	40.35	300.0	88.88
	A.2	Syringomyelia + otitis	Yes	5	204.9	68.0	475.5	161.0
			No	1	113.6	113.6	113.6	0
	A.3	Syringomyelia + concomitant neurological disease	Yes	3	249.1	54.79	521.5	242.9
			No	4	113.4	66.81	169.1	43.09
C	Healthy control group		Yes	/	/	/	/	/
			No	8	186.2	98.63	309.6	186.2

pg/ml: picograms per milliliter

**Fig. 4:**
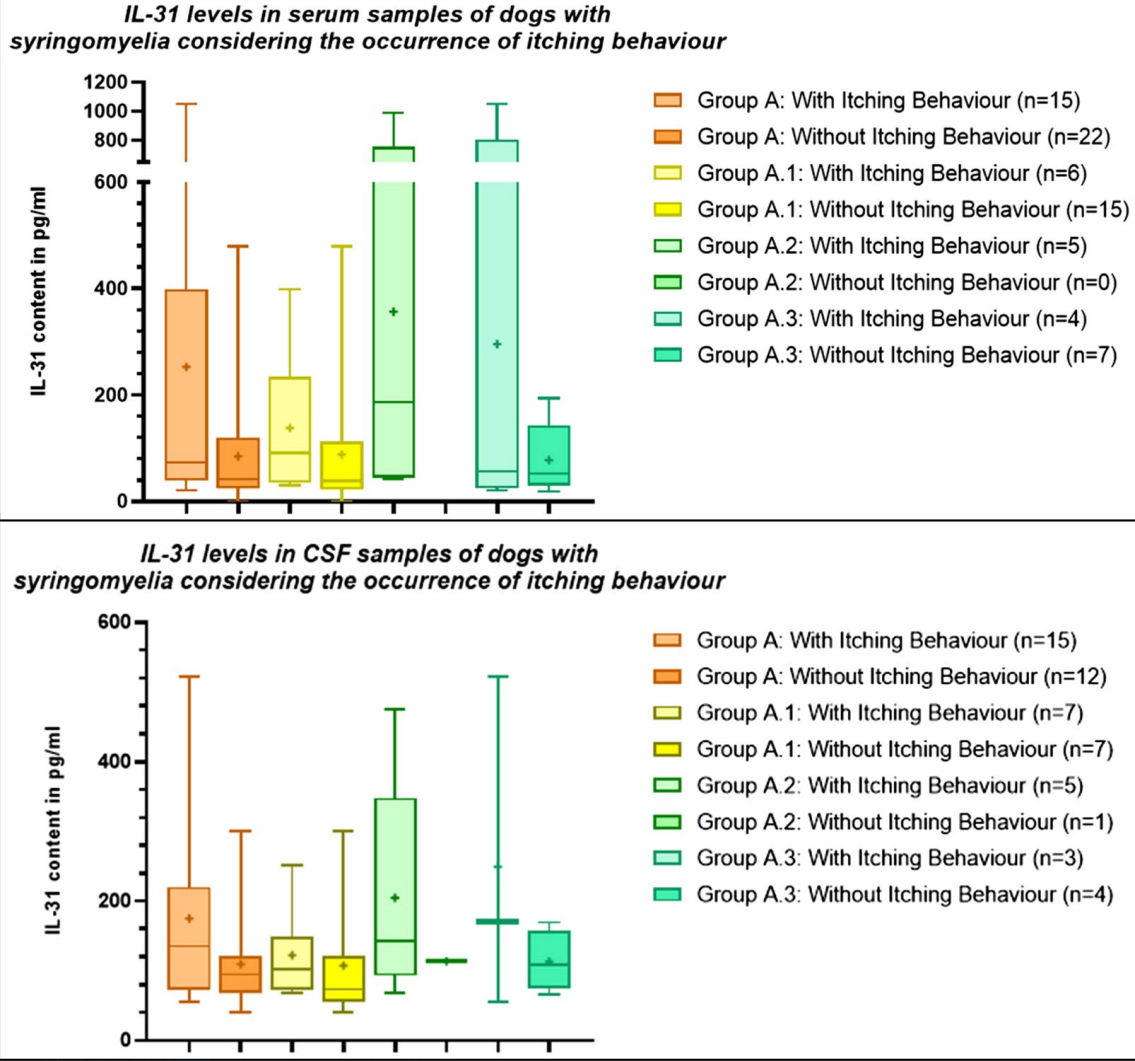
Interleukin-31 (IL-31) levels in serum and cerebrospinal fluid (CSF) samples of dogs with syringomyelia considering their itching behaviour. The groups did not differ significantly (p > .05). The boxplots show whiskers from the minimum to maximum and boxes from the 25th to 75th percentiles as well as the median (horizontal line) and the mean (+). Group A: Patients with syringomyelia total; group A.1: patients with syringomyelia only; group A.2: patients with syringomyelia + otitis; group A.3: patients with syringomyelia + concomitant neurological disease; pg/ml: picograms per milliliter

In order to rule out an influence of otitis or concomitant neurological disease on itching behaviour [22] and IL-31 levels, difference between IL-31 levels in serum and CSF of dogs with and without itching behaviour were compared separately in dogs with syringomyelia only (group A.1), with syringomyelia and otitis (group A.2), and dogs with syringomyelia and concomitant neurological disease (A.3) respectively. No significant difference between IL-31 levels in serum or CSF was detected in dogs with and without itching behaviour for each group (p > .05, Fig. 4). When comparing the groups, the mean IL-31 content in the serum was higher in dogs with syringomyelia and additional otitis showing itching behaviour (group A.2) in comparison to the other dogs with syringomyelia displaying itching behaviour from group A.1 + A.3 (Table 2). In CSF samples the IL-31 content was highest in dogs with concomitant neurological diseases (group A.3) showing itching behaviour followed by dogs with syringomyelia and otitis (group A.2) and itching behaviour (Table 2).

### Pain

To evaluate if pain is correlated with elevated IL-31 levels in dogs with syringomyelia (group A), statistical evaluation of this aspect was performed. Of the 48 patients with syringomyelia, 25 patients showed signs of pain in the general or neurological examination or in the history (serum n = 17/39, CSF n = 17/27) and in 17 patients no signs of pain were detected (serum n = 17/39, CSF n = 8/27). No information regarding signs of pain was available for 6 patients (serum n = 5/39, CSF n = 2/27). Of the dogs with syringomyelia and concomitant neurological disease with signs of pain, 4 dogs had intervertebral disc herniation, and one of each suffered from trauma, infarct, or nerve sheath tumour.

**Fig. 5:**
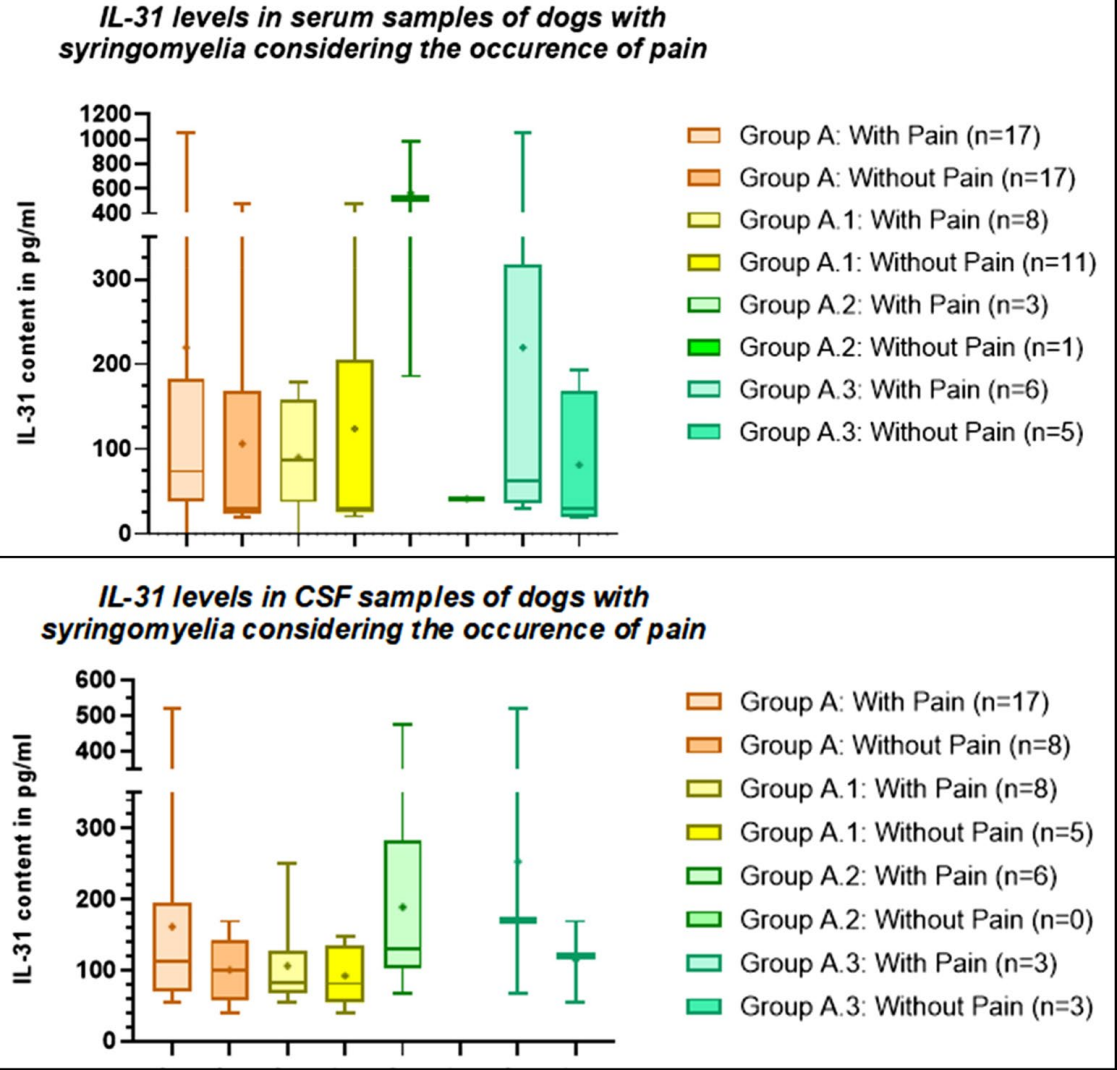
Interleukin-31 (IL-31) levels in serum and cerebrospinal fluid (CSF) samples of dogs within the syringomyelia subgroups divided by the presence of signs of pain. The differences were not significant (p>.05). The boxplots show whiskers from the minimum to maximum and boxes from the 25th to 75th percentiles as well as the median (horizontal line) and the mean (+). Group A: Patients with syringomyelia total; group A.1: patients with syringomyelia only; group A.2: patients with syringomyelia + otitis; group A.3: patients with syringomyelia + concomitant neurological disease. pg/ml: picograms per milliliter

Overall, the mean IL-31 level in patients with syringomyelia, and signs of pain was nearly about twice as high as the mean IL-31 value in the serum of patients with syringomyelia without pain (Table 3), but the difference was not statistically significant (p = .1061, Fig. 5). No significant difference was found in IL-31 levels in CSF between dogs with syringomyelia (group A) with and without signs of pain (p = .2351, Fig. 5).

Since pain can occur due to otitis and other neurological disease like intervertebral disc protrusion or extrusion [23] or neoplasia [24], difference between IL-31 levels in serum and CSF of dogs with and without signs of pain were compared separately in dogs with syringomyelia only (group A.1), with syringomyelia and otitis (group A.2), and dogs with syringomyelia and concomitant neurological disease (A.3) (Table 3). Mean IL-31 levels in serum of dogs with signs of pain tended to be higher than in dogs without signs of pain in the group of dogs with syringomyelia and otitis (group A.2) and in the serum and CSF in the group of dogs with syringomyelia and concomitant neurological disease (group A.3), although the difference was not statistically significant (Fig. 5).

**Table 3:** Descriptive statistics of Interleukin-31 (IL-31) level in serum and cerebrospinal fluid (CSF) samples of dogs with syringomyelia considering the occurrence of signs of pain

Group			Signs of Pain	Number	Mean IL-31 level (pg/ml)	Minimum IL-31 level (pg/ml)	Maximum IL-31 level (pg/ml)	Standard deviation
**Serum**								
A	Syringomyelia total		Yes	17	219.5	0	≥ 1050	323.5
			No	17	106.5	19.2	478.8	139.6
	A.1	Syringomyelia only	Yes	8	89.9	0	179.0	65.68
			No	11	123.9	20.53	478.8	165.7
	A.2	Syringomyelia + otitis	Yes	3	564.7	186.1	986.2	401.8
			No	1	41.29	41.29	41.29	0
	A.3	Syringomyelia + concomitant neurological disease	Yes	6	219.6	29.54	≥ 1050	407.2
			No	5	81.24	19.2	193.7	81.56
C	Healthy control group		Yes	/	/	/	/	/
			No	10	80.73	42.67	137.2	37.87
**CSF**								
A	Syringomyelia total		Yes	17	161.6	54.98	521.5	138.5
			No	8	100.7	40.35	169.1	46.1
	A.1	Syringomyelia only	Yes	8	106.1	54.98	251.4	63.80
			No	5	94.34	40.35	148.5	42.93
	A.2	Syringomyelia + otitis	Yes	6	189.7	68.0	475.5	148.7
			No	0	/	/	/	/
	A.3	Syringomyelia + concomitant neurological disease	Yes	3	253.1	66.81	521.5	238.2
			No	3	114.6	54.79	169.1	57.36
C	Healthy control group		Yes	/	/	/	/	/
			No	8	186.2	98.63	309.6	186.2

pg/ml: picograms per milliliter

## Discussion

The purpose of this study was to investigate the correlation between IL-31 levels in serum and CSF in patients with syringomyelia and itching behaviour. We hypothesized that dogs with syringomyelia and associated sings of pain or increased itching behaviour have higher levels of IL-31 in CSF and/or serum. It could be shown that individual patients from group A (patients with syringomyelia) had increased, but not significant higher IL-31 levels in the serum and CSF. Although the difference is not significant either, dogs displaying itching behaviour tend to have higher mean IL-31 levels in serum and CSF. Especially dogs with additional otitis (group A.2) or intervertebral disc herniation (group A.3) display higher IL-31 values comparable to control dogs with atopic dermatitis (group B), while dogs with syringomyelia only (group A.1) tend to have IL-31 levels comparable to healthy control beagles (group C).

These results suggest that increased levels of IL-31 in serum of single dogs with syringomyelia might not be driven by the syringomyelia itself but by inflammatory diseases or an inflammatory reaction to any other underlying disease. Although not studied extensively, major inflammatory components are not described in the pathogenesis of syringomyelia [14]. Small numbers of macrophages and variable degree of gliosis with increased numbers of microglia, and only sporadically perivascular infiltration with lymphocytes are found in histopathology [25, 26]. In contrast, in the spinal cord of dogs with intervertebral disc herniation significant inflammatory reaction is present [27]. Despite the trauma caused to the spinal cord and adjacent tissue by the herniated disc material, the disc material itself can cause an immune-mediated reaction directed against the extruded tissue [28, 29], with a T-helper cell mediated inflammatory reaction [30, 31]. IL-31 is mostly produced by T-helper cells [6, 9]. This circumstance could explain the higher levels of IL-31 values in serum or CSF samples of some dogs with intervertebral disc herniation in the current study.

IL-31 levels of serum and CSF seem to be correlated, but the correlation between them remains unclear. However, increased intrathecal production of IL-31, or passage of IL-31 through a possible damaged blood-CSF barrier can be considered. Due to its retrospective nature, the integrity of the blood-CSF barrier was not evaluated [32].

Of 48 dogs with syringomyelia 8 dogs had additional ear disease (suspected primary secretory otitis media or infectious otitis media and interna). Atopic dermatitis frequently causes otitis externa and media in dogs [33, 34]. Therefore, higher levels of IL-31 in serum and CSF in these dogs are more likely the result of a possible, undiagnosed underlying atopic dermatitis and less likely due to syringomyelia. Itching in the ear and neck area is also described as a clinical sign of ear diseases, so that the itching behaviour in patients from group A.2 might be rather a result of otitis and not caused by syringomyelia itself. Canine atopic dermatitis is mostly driven by a Th2 response, one key factor driving pruritus [3, 6].

In a previous study by Gonzales et al., only 57% of dogs with atopic dermatitis had increased IL-31 levels [4]. Indeed, in comparison only a part of dogs with syringomyelia and otitis or concomitant neurological disease and increased itching behaviour had increased IL-31 levels in the present study. Three out of five dogs from group A.2 (dogs with syringomyelia and otitis) and three of seven dogs with intervertebral disc herniation had IL-31 levels above the one of the healthy control group, which is in line with the results of Gonzales et al. [4].

Itching behaviour was not significantly correlated with increased IL-31 levels in serum of dogs with syringomyelia. Several pathways for itching behaviour and pruritogens are described besides IL-31 [1, 11, 35]. Especially in dogs with syringomyelia so called air-scratching is observed. Here, dogs make excessive scratching movements, but the paw does not touch the skin [15, 19]. It was suggested, that syringomyelia causes damage to descending inhibitory pathways and the resulting disinhibition of lumbar central pattern generators cause clinical signs of air-scratching without the real feeling of itch [19]. In contrast, in atopic dermatitis, the animals typically show itching with skin contact and sometimes even automutilation [36]. Due to the retrospective nature of the study it could not be evaluated if dogs showed air-scratching or “real” itching. Repeated administration of IL-31 leads to increased pruritus, an increased expression of IL31-RA and OSMR in the dorsal root ganglion [37] and at the same time to an increase in small sensory neurons in the dorsal root ganglion [38]. As a result, IL-31 increases the sensitivity to minimal stimuli leading to itching [38]. This upregulation could be one reason for the mostly progressive clinical course of pruritus in atopic dermatitis [36], while air-scratching in the context of syringomyelia is typically not progressive, but can be triggered by exercise and excitement in some dogs [15].

As pain and dysesthesia are other clinical signs of dogs with syringomyelia [13, 14] and pruritus and pain share the same central pathways [8, 11, 12], we also examined the correlation between pain and IL-31 levels in serum. Although, no significant difference between dogs with and without signs of pain could be shown, those dogs with otitis and dogs with signs of pain tended to have higher serum IL-31 levels than dogs without sign of pain. In inflammation, chemical factors and chemokines produced during tissue damage lower the threshold of afferent sensory neurons and also alter central pain sensation [39]. It is likely that increased IL-31 levels do not cause pain but are rather a bystander effect of inflammatory diseases.

In syringomyelia, signs of pain are most probably the result of central disinhibition of excitatory neurons secondary to damage of lamina I and II of the dorsal horn [12], as pain and dysesthesia is associated with asymmetrical involvement of the dorsal horns of the spinal cord grey matter [12]. Although IL-31 receptors are found in the dorsal horn of the spinal cord [16], we could not show an involvement of IL-31 in the pathogenesis of pain in syringomyelia.

## Conclusion

In summary, itching behaviour or signs of pain in dogs with syringomyelia seem not to be caused by increased IL-31 levels in serum and CSF. In individual patients, where syringomyelia was accompanied by otitis media and/or interna, or intervertebral disc disease, higher IL-31 levels were measured in serum and CSF. Although, there was no statistical difference, this might have been caused by the concurrent T-helper cell mediated inflammatory reaction of these underlying diseases.

## Materials and methods

This study was conducted as a retrospective multicentric study. Biobanks were searched for archived serum and CSF samples from dogs with syringomyelia or atopic dermatitis presented between 2010 and 2021 in the Department of Small Animal Medicine and Surgery, University of Veterinary Medicine Hannover (Hannover, Germany), the Queen Mother Hospital for Animals of the Royal Veterinary College (London, United Kingdom) and the Department of Small Animal Medicine, University of Leipzig (Leipzig, Germany). All patient samples were obtained as part of routine diagnostic examination with owner’s informed consent. The serum and CSF samples from healthy beagles were residuals of previous studies performed according to the ethical guidelines of the University of Veterinary Medicine Hannover and approved by the Lower Saxony State Office for Consumer Protection and Food Safety (Lower Saxony, Germany; animal experiment reference number 33.12-42502-04-20/3352). All samples were stored after sampling at -80 °C.

General information about breed, age, sex as well as specific information about dogs including clinical signs with their onset and duration, signs of pain and/or pruritus, previous treatment, possible other comorbidities which might cause itching behaviour and results of the examinations and diagnostic tests including general and neurological examination, blood examination, MRI of the cervical/thoracic/lumbar spinal cord and CSF tap were taken from the electronic medical record of each individual patient.

**Fig. 6:**
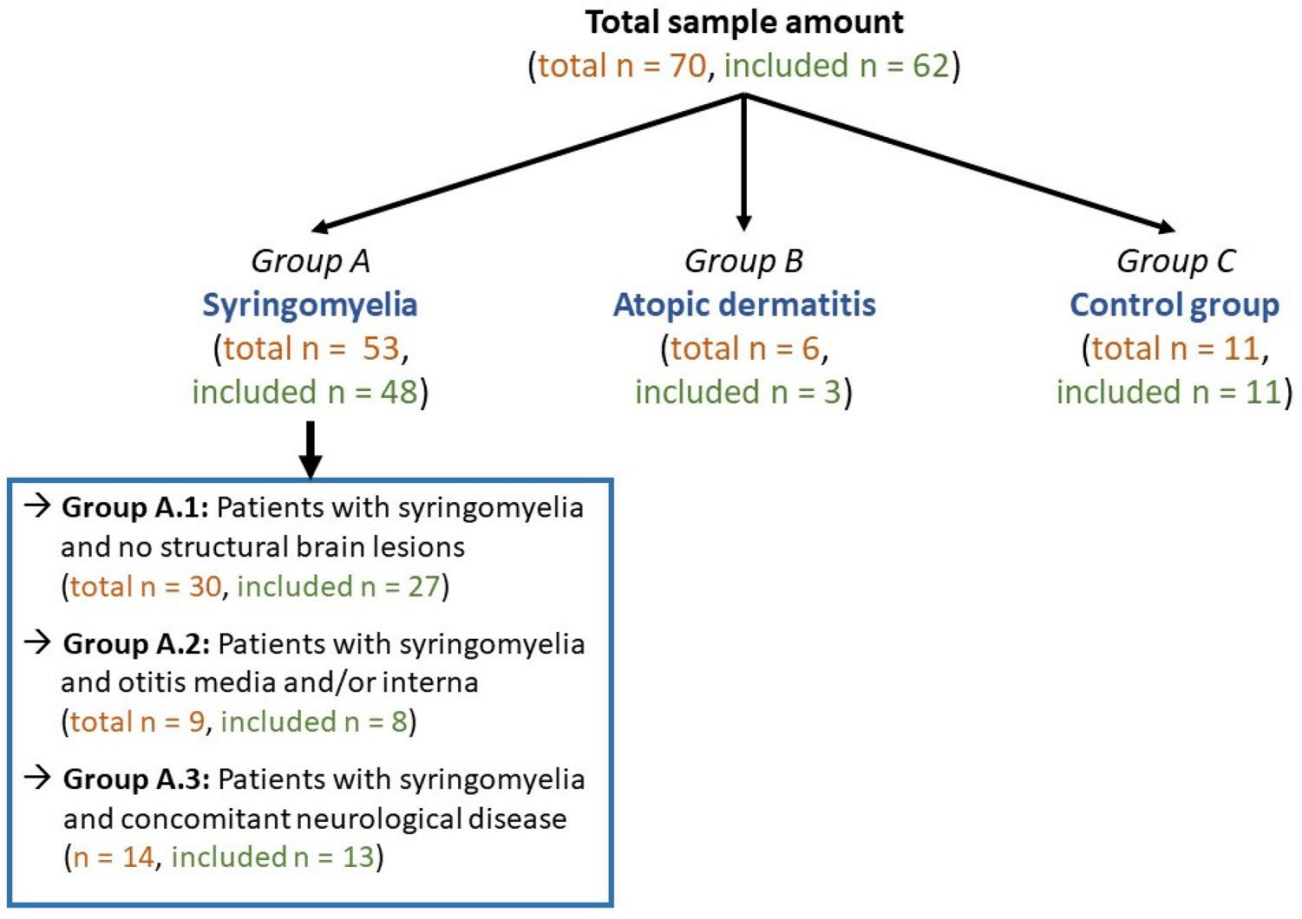
Overview about grouping of all samples. The total amount of measured samples is marked in orange colour. Some samples had to be excluded after measuring IL-31 due to a coefficient of variation higher than 20%. All included samples were marked in green colour

The included serum and CSF samples of the dogs were grouped according to their underlying diseases into three groups (Fig. 6):

- Group A: Patients with syringomyelia (n = 48). The diagnosis of syringomyelia was made on the basis of MRI of at least the cervical or thoracic spinal cord, based on previously published criteria [14]. No further differentiation between localisation or the size of the syringomyelia was performed in this study.
- Group B: Patients with atopic dermatitis (n = 3). Diagnosis of atopic dermatitis was based on signalment, indoor lifestyle, affected areas to be at the level of feet and pinnae, and exclusion of other possible causes like cutaneous infections or ectoparasites [36]. For these patients only serum samples were available and included in this study.
- Group C: Healthy control group (n = 11). This group includes samples from healthy clinic owned beagles. The general clinical examination was unremarkable in these dogs and all included dogs showed no clinical signs for atopic dermatitis.

Group A, patients with syringomyelia, was further divided into three subgroups considering the presence of concomitant diseases.

- Patients with syringomyelia and no structural brain lesions other than ventriculomegaly and hydrocephalus were defined as subgroup A.1 (n = 27). This diagnosis was made based on unremarkable MRI studies of the head and/or cervical/thoracic spinal cord except for syringomyelia and a possible enlargement of the ventricles. The routine CSF examination of these patients was without pathological findings.
- Group A.2 included patients with syringomyelia and otitis media and/or interna (n = 8). Primary secretory otitis media (PSOM, n = 4) or infectious otitis media and interna (n = 4) were diagnosed based a combination of MRI findings (n = 7), otoscopic examination (n = 6), cytologic examination of material sampled from the middle ear by myringotomy (n = 6) and/or microbiological cultures (n = 5). Dogs with abnormal signal intensities and/or pathological contrast enhancement of the brain and/or the meninges in MRI and/or abnormal CSF findings were excluded.
- Group A.3 included patients with syringomyelia and concomitant neurological disease – mostly structural brain or spinal cord lesion other than hydrocephalus (n = 13).

Presence of pruritus was considered, if either owner’s description and/or physical as well as neurological examination performed by a board-certified neurologist or a neurologist-on-training revealed signs of increased itching behaviour or air-scratching, defined as recurring, scratching movements in the direction of the neck, without skin contact according to the definition of Rusbridge et al. [13, 15].

Pain was considered, if either owner’s description and/or physical as well as neurological examination performed by a board-certified neurologist or a neurologist-on-training revealed pain face or spontaneous vocalization when touched or palpated neck region. Additionally, if owner recorded reduced activity or lethargy, refusal to jump or walk stairs, sleeping with abnormally elevated head posture, and changes in emotional behaviour, such as reduced greeting or increased aggression [15, 40], pain was considered as well.

The obtained CSF and serum samples were examined using a competitive Enzyme-Linked Immunosorbent Assay (ELISA; Canine Interleukin 31 ELISA kit, MyBioSource, Inc., San Diego, USA, catalog number: MBS740462). The frozen samples were thawed immediately before the assay was performed and brought to room temperature according to the manufacturer’s instruction. Subsequently, the ELISA was carried out according to the enclosed instructions of the manufacturer. The measurement of the optical density of the samples was carried out immediately after the addition of the stop solution from the ELISA kit in a Multi-Detection Microplate Reader (Synergy^™^ 2, BioTek Instruments, Inc., Winooski, Vermont, USA) at 450 nm. Each sample was measured as duplicates in the same test and the coefficient of variation was determined.

Only results from samples with a coefficient of variation below 20% were considered for the statistical analysis (Fig. 6). The coefficient of variation is used as a marker for the intraassay variation for testing in duplicates of each sample [41]. If the coefficient of variation-values were higher than 20% for one sample, the measurement was repeated. This was not possible for all samples due to a small sample size. If the coefficient of variation-value was again higher than 20% in the second circle or if the sample amount was too small for repeated measurements, these samples were excluded from statistical analysis (Fig. 6). A standard reference curve was established by the manufacturer for the ELISA using six standard solutions with a specified canine IL-31 level starting from 0 to 1000 pg/ml. If IL-31 values exceeded the upper detection range of 1050 pg/ml, values are given as > 1050 pg/ml and statistical analysis was performed with the fixed value of 1050 pg/ml.

The statistical evaluation was carried out using the GraphPad Prism 9 software (GraphPad Software [part of Dotmatics], San Diego, California, USA). After evaluating descriptive statistics, the examination for normal distribution was carried out using the Shapiro-Wilk test. The results of CSF IL-31 levels were logarithmised to achieve a normal distribution to carry out further statistical tests. Afterwards the Mann Whitney or Kruskal-Wallis test were used to analyse skewed data. The unpaired t-test and the one-way analysis of variance (ANOVA) were performed with normal distributed data. A significance level p < .05 was considered for statistical analysis. In paired serum and CSF samples the statistical evaluation of correlation was performed with the Pearson correlation coefficient.

### Electronic supplementary material

Below is the link to the electronic supplementary material.

Supplementary Material 1

## Data Availability

The datasets generated, used and analysed during the current study are available from the corresponding author on reasonable request.

## List of abbreviations

CSF: Cerebrospinal fluid
ELISA: Enzyme-Linked Immunosorbent Assay
IL-31: Interleukin-31
IL-31RA: IL-31 receptor A
ml: Millilitre
MRI: Magnet Resonance Imaging
nm: Nanometer
OSMR: Oncostatin M receptor β
pg: Picogram
Th2: T-helper 2
