# Supplementary material for: Interleukin-31 in serum and cerebrospinal fluid of dogs with syringomyelia
Source: BMC Vet Res. 2023 Nov 23;19:244. doi: 10.1186/s12917-023-03817-8 (PMC10666301; doi:10.1186/s12917-023-03817-8)
Supplement: Supplementary file 1 — Supplementary Material 1 [file 12917_2023_3817_MOESM1_ESM.pdf]

# **Supplement 1:** Distribution of serum and CSF samples

| Group | | Total | Serum | CSF |
|---|---|---|---|---|
| **A** | Syringomyelia total | 48 | 39 | 27 |
| **A.1** | Syringomyelia only | 27 | 23 | 14 |
| **A.2** | Syringomyelia + otitis | 8 | 5 | 6 |
| **A.3** | Syringomyelia + concomitant neurological disease | 13 | 11 | 7 |
| **B** | Atopic dermatitis | 3 | 3 | 0 |
| **C** | Healthy control group | 11 | 10 | 8 |
| | Total | 62 | 52 | 35 |

## Itching behaviour

| Itching behaviour in group A (Syringomyelia total) | Total | Serum | CSF |
|---|---|---|---|
| With itching behaviour | 21 | 15 | 15 |
| Without itching behaviour | 25 | 22 | 12 |
| Unknown itching behaviour | 2 | 2 | 0 |
| Total | 48 | 39 | 27 |

**Divided in subgroups**

| Itching behaviour in subgroup A.1 (Syringomyelia only) | Total | Serum | CSF |
|---|---|---|---|
| With itching behaviour | 10 | 6 | 7 |
| Without itching behaviour | 15 | 15 | 7 |
| Unknown itching behaviour | 2 | 2 | 0 |
| Total | 27 | 23 | 14 |

| Itching behaviour in subgroup A.2 (Syringomyelia + otitis) | Total | Serum | CSF |
|---|---|---|---|
| With itching behaviour | 7 | 5 | 5 |
| Without itching behaviour | 1 | 0 | 1 |
| Unknown itching behaviour | - | - | - |
| Total | 8 | 5 | 6 |

| Itching behaviour in subgroup A.3 (Syringomyelia + concomitant neurological | Total | Serum | CSF |
|---|---|---|---|
| With itching behaviour | 4 | 4 | 3 |
| Without itching behaviour | 9 | 7 | 4 |
| Unknown itching behaviour | - | - | - |
| Total | 13 | 11 | 7 |

## Signs of Pain

| Signs of pain in group A (Syringomyelia total) | Total | Serum | CSF |
|---|---|---|---|
| With signs of pain | 25 | 17 | 17 |
| Without signs of pain | 17 | 17 | 8 |
| Unknown sings of pain | 6 | 5 | 2 |
| Total | 48 | 39 | 27 |

**Divided in subgroups**

| Presence of pain in subgroup A.1 (Syringomyelia only) | Total | Serum | CSF |
|---|---|---|---|
| With signs of pain | 12 | 8 | 8 |
| Without signs of pain | 11 | 11 | 5 |
| Unknown sings of pain | 4 | 4 | 1 |
| Total | 27 | 23 | 14 |

| Presence of pain in subgroup A.2 (Syringomyelia + otitis) | Total | Serum | CSF |
|---|---|---|---|
| With signs of pain | 6 | 3 | 6 |
| Without signs of pain | 1 | 1 | 0 |
| Unknown sings of pain | 1 | 1 | 0 |
| Total | 8 | 5 | 6 |

| Presence of pain in subgroup A.3 (Syringomyelia + concomitant neurological disease) | Total | Serum | CSF |
|---|---|---|---|
| With signs of pain | 7 | 6 | 3 |
| Without signs of pain | 5 | 5 | 3 |
| Unknown sings of pain | 1 | 0 | 1 |
| Total | 13 | 11 | 7 |

**Supplement 1:** Distribution of serum and CSF samples

In this study, dogs with magnetic resonance imaging (MRI) confirmed syringomyelia (group A) without (subgroup A.1.) or with other comorbidities such as otitis (subgroup A.2) or further concomitant neurological diseases such as intervertebral disc extrusion or neoplasia (group A.3) were included. Healthy dogs and dogs with atopic dermatitis served as control groups. The Interleukin-31 levels in serum and CSF samples of these dogs were measured

Increased itching behaviour or signs of pain were documented in dogs with syringomyelia separately for each subgroup, to examine a possible influence on the Interleukin-31 level in the samples.

CSF: cerebrospinal fluid
